# Supplementary material for: Learning curve and surgical outcome of robotic assisted colorectal surgery with ERAS program
Source: Sci Rep. 2022 Nov 29;12:20566. doi: 10.1038/s41598-022-24665-w (PMC9709162; doi:10.1038/s41598-022-24665-w)
Supplement: Supplementary file 2 — Supplementary Information 2. [file 41598_2022_24665_MOESM2_ESM.zip › RAL Raw data-Ñ[▒K/135/OP N_ñw╝╨░O▒Kñσ.pdf]

W52 / W52

臺中榮民總醫院
手術室護理記錄

[redacted]

基本資料

- 室別： 開刀房-17室，手術科別： CRS，手術類別： 常規，日期： 2021/12/18
- 進入時間： 0810 ； 麻醉開始時間： 0825 ； Time Out時間： 1035 ； 劃刀時間： 1036 ； 手術結束時間： 1405 送出時間： 1440 ；
- 麻醉方式： GE
  診斷：S colon tumor 15 cm FAV
  手術名稱：Robot Assisted Colectomy(L)
  手術局部用藥：NIL
  術前預防性抗生素：
  - 抗生素名稱一： Veterin 1GM IVD由麻醉人員給予
  - 時間： 0925
  - 上次簽章: 林靖蓉

  ，追加預防性抗生素：
- 手術醫師： 林俊余
- 麻醉醫師： VS 陳永銘
- 刷手護士A ： 劉雅汶，時間： 0810，接替刷手一： 林裕瑋，時間： 1300
  流動護士A ： 林靖蓉 ，時間： 0810 ，流動護士B ： 沈佳融 ，時間： 0810 ，接替流動一： 劉雅汶 ，時間： 1330

皮膚狀況

A-V Shunt： 無
術前： 完整
記錄者： 上次簽章: 林靖蓉
術後： 完整
記錄者： 劉雅汶

手術準備

手術臥式 單一手術臥式

-
  - 手術臥式： 截石臥式
  - 使用附件： 腳架 ，其它： latteral support、灰色頭枕
  - 皮膚準備： 10%Aq Betadine ，Hibiscrub ，2% Chlorhexidine
  - 電燒： 使用電燒: 電極片位置： 其它： 電燒迴路軟墊 ，雙極電燒 ，記錄者： 上次簽章: 林靖蓉

止血帶T： 無

植入物

- ENDO GIA 45綠釘*2 /S-colon 切除處

，記錄者: 上次簽章: 沈佳融

標本送檢

- 細菌培養 無
- 細胞學 無
- 冰凍切片 無
- 病理標本
  組織名稱一：S-colon
  ，
  組織名稱二：distal cut end
  ，
  組織名稱三：IMA roots
- 截肢 無

標本交付 標本： 3 袋 ，標本： 0 盒 ，標本： 0 瓶 ，細胞學： 0 管 ，Culture Tube： 0 管 ，被交付醫師： R 張育豪 ，

- 記錄者： 劉雅汶
- 時間： 1430

傷口術後敷料

傷口敷料： 傷口敷料： 無

- 請於圖上標示部位：

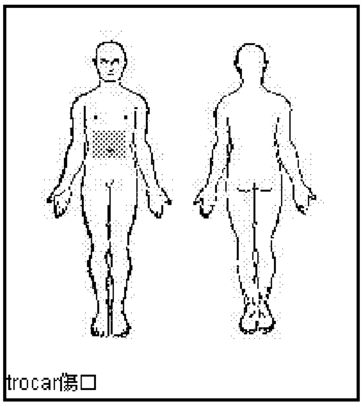

- 傷口敷料： 軟性敷料

導管

導管	天數	導管資訊	插入日期	插入時間	更換日期	拔除日期	拔除時間	拔除原因	預計拔除/更換	預計拔除/更換日期
Double Lumen CVC(1)	1	號碼:8Fr ; 深度:15cm; 部位:jugular (右); 象限:RU;	2021/12/18	0850						
A-line (1)	1	號碼:20G ; 部位:左手;	2021/12/18	0900						
Sump NG Tube(1)	1	號碼:16 Fr.; 深度:53cm; 部位:胃;	2021/12/18	0830						
Epidural PCA(1)	1	部位:硬脊膜下;	2021/12/18	0840						
Foley(1)	1	號碼:16 Fr.; 部位:膀胱;	2021/12/18	0910						

用物計數

- 手術中出血量： minimal c.c.
- 手術中尿量： 250 c.c.
-
    - 其它： 無

用物計數： 多切口同時縫合

- 1.器械： 正確，2.縫針： 正確，3.刀片： 正確，4.紗布： 正確，5.腹部墊： 未使用，6.紗球： 未使用，7.PEANUTS ： 未使用，8.紗條： 正確，9.絲帶： 未使用，10.針頭： 未使用，11.Vessel loop： 未使用，12.Boots： 未使用，13.Jelco： 未使用，14.棉條： 未使用，
    - 第一次：
        - 刷手護士 ： 林裕瑋
        - 流動護士： 上次簽章: 沈佳融
        - 時間： 1330

    ,
    - 第二次：
        - 刷手護士 ： 林裕瑋
        - 流動護士： 上次簽章: 沈佳融
        - 時間： 1340

    ,
    - 第三次：
        - 刷手護士 ： 林裕瑋
        - 流動護士： 上次簽章: 沈佳融
        - 時間： 1350

備註

備註： No.2 0度 Davinci scopy

- 記錄者： 上次簽章: 林靖蓉
- 時間： 0830

填寫護理人員 劉雅汶

正式摘要

W52 / W52

doctorRank:B
